# Solving the cooling flow problem with combined jet-wind AGN feedback

**DOI:** 10.1126/sciadv.aed6394

**Published:** 2026-07-03

**Authors:** Aoyun He, Feng Yuan, Suoqing Ji, Minhang Guo, Yuan Li, Haiguang Xu, Ming Sun, Haojie Xia, Yuanyuan Zhao

**Affiliations:** ^1^ Shanghai Astronomical Observatory, Chinese Academy of Sciences, 80 Nandan Road, Shanghai 200030, China.; ^2^ University of Chinese Academy of Sciences, No. 19A Yuquan Road, Beijing 100049, China.; ^3^ Center for Astronomy and Astrophysics and Department of Physics, Fudan University, Shanghai 200438, China.; ^4^ Key Laboratory of Nuclear Physics and Ion-Beam Application (MOE), Fudan University, Shanghai 200433, China.; ^5^ ShanghaiTech University, 393 Middle Huaxia Road, Shanghai 201210, China.; ^6^ Department of Astronomy, University of Massachusetts, 710 North Pleasant Street, Amherst, MA 01003, USA.; ^7^ State Key Laboratory of Dark Matter Physics, School of Physics and Astronomy, Shanghai Jiao Tong University, 800 Dongchuan Road, Shanghai 200240, China.; ^8^ Department of Physics & Astronomy, University of Alabama in Huntsville, Huntsville, AL 35899, USA.

## Abstract

Active galactic nucleus (AGN) feedback is widely viewed as the most promising solution to the long-standing cooling flow problem in galaxy clusters, yet previous models prescribe jet properties inconsistent with accretion physics. We perform an idealized hydrodynamic simulation of a galaxy cluster with no merger history and a relaxed state, with its other properties similar to the Perseus cluster using the MACER framework, incorporating both jets and winds whose properties are constrained by general relativistic magnetohydrodynamic simulations of black hole accretion and observations. The combined feedback reproduces key observables, including cold gas mass, star formation rate, thermodynamic radial profiles, and black hole growth, while jet-only or wind-only models fail. The success arises from turbulence driven by jet-wind shear that enhances kinetic-to-thermal energy conversion, boosting heating efficiency by factors of 3 and 6 relative to wind-only and jet-only cases, respectively.

## Teaser

Combined wind and jet feedback self-consistently solves the cooling flow problem in galaxy clusters.

## INTRODUCTION

A large fraction of galaxy clusters exhibits a cool-core structure, where the central gas is characterized by high density and low entropy, with cooling timescales on the order of ∼1 Gyr (billion years) (*1*). Under such conditions, a strong cooling inflow at rates of $10^{2}\;\text{to}\;10^{3}\;\text{solar mass}\;\left(M_{⊙}\right)$ ${\text{year}}^{-1}$ is expected to develop, accompanied by the accumulation of large amounts of cold gas ($∼10^{10}\;\text{to}\;10^{11}\;M_{⊙}$) in cluster cores and high star formation rates (SFRs) of hundreds or even thousands of $M_{⊙}$ per year in the central brightest cluster galaxies (BCGs) (*2*). However, multiband observations have found far less cold gas ($∼10^{8}\;\text{to}\;10^{10}\;M_{⊙}$) and smaller SFR ($0.1\;\text{to}\;10s\;M_{⊙}$ ${\text{year}}^{-1}$) than predicted (*3*). This discrepancy is referred to as the “cooling flow problem.”

To reconcile this discrepancy, many mechanisms have been proposed, such as stellar and supernova feedback (*4*), cosmic rays (*5*), magnetic fields (*6*), and thermal conduction (*7*). Among them, active galactic nucleus (AGN) feedback has emerged as the dominant solution (*8*–*20*), supported by strong observational evidence linking AGN activity to suppressed cooling, x-ray cavities, and multiphase gas in low-entropy cores (*21*–*23*). However, the AGN feedback models adopted in existing simulations commonly suffer from important physical drawbacks.

First, because of limited resolution, almost all simulations cannot resolve the outer boundary of the accretion flow, commonly referred to as the Bondi radius, where the gravitational energy of the gas is comparable to its thermal energy; thus, the black hole accretion rate cannot be reliably calculated (*24*, *25*). Second, with jets (or winds) being invoked to be the main component of AGN feedback, their parameters are usually treated as free, including the opening angle, velocity, and mass flux (*9*, *12*, *15*), and the adopted parameter values are often inconsistent with the theories of black hole accretion and jet formation. Even when the jet parameters are treated as free, some studies adopt jet precession or reorientation to enhance the interaction with the intracluster medium (ICM) (*11*, *14*, *18*). Third, although theoretical and observational works have established that winds are generally expected to be present when jets are launched (*26*–*30*), most studies to date have not simultaneously and self-consistently incorporated both jet and wind. Last, many existing idealized cooling flow simulations [e.g., (*13*, *14*)] focus primarily on addressing the cooling flow problem itself without examining whether the proposed solutions are also consistent with other observational constraints, such as the growth of the black hole, which is more commonly tracked in cosmological simulations [e.g., (*31*)].

## RESULTS

### Simulations of the evolution of a Perseus-like cluster with MACER

**Fig. 1.**
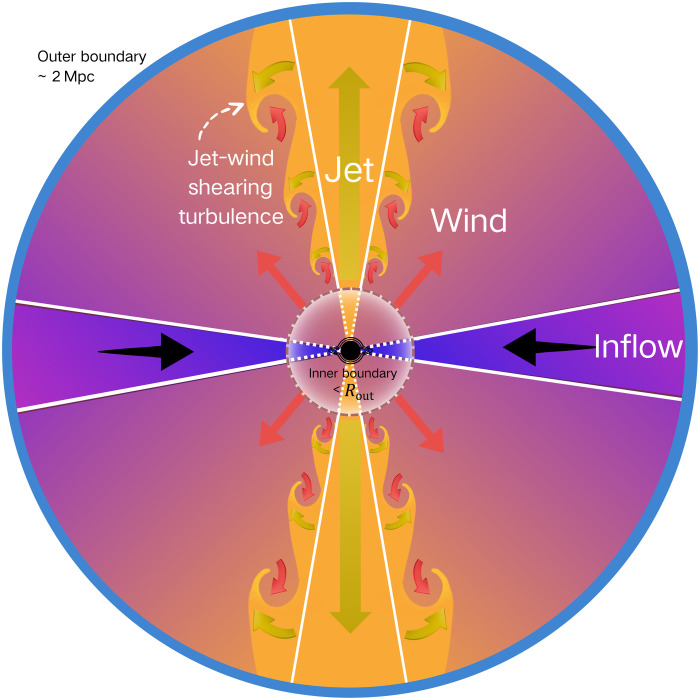
Schematic of the fiducial JetWind model. The dashed circle marks the simulation’s inner boundary, located within the outer boundary of the accretion flow $R_{\text{out}}$, enabling self-consistent calculation of the black hole accretion rate and accurate coupling of AGN outputs to the ICM. Jet and wind parameters are taken from GRMHD simulations. Shear between them triggers Kelvin-Helmholtz instabilities that drive turbulence, whose dissipation converts jet kinetic energy into ICM heat, suppressing the cooling flow.

Here, we perform idealized high-resolution three-dimensional (3D) hydrodynamic simulations of the evolution of a galaxy cluster with no merger history, no cosmological inflow, and a relaxed state, with its other properties similar to the Perseus cluster. The simulations are based on the MACER framework, a model we have developed to investigate the evolution of a single galaxy, focusing on the role of AGN feedback [(*32*, *33*); see Materials and Methods and https://macer-project.github.io for details of the simulations]. In addition to AGN feedback, other important physical processes such as star formation, stellar feedback, and radiative heating and cooling of the ICM by the AGN are also included in MACER. A schematic diagram of the simulation is shown in Fig. 1.

**Fig. 2.**
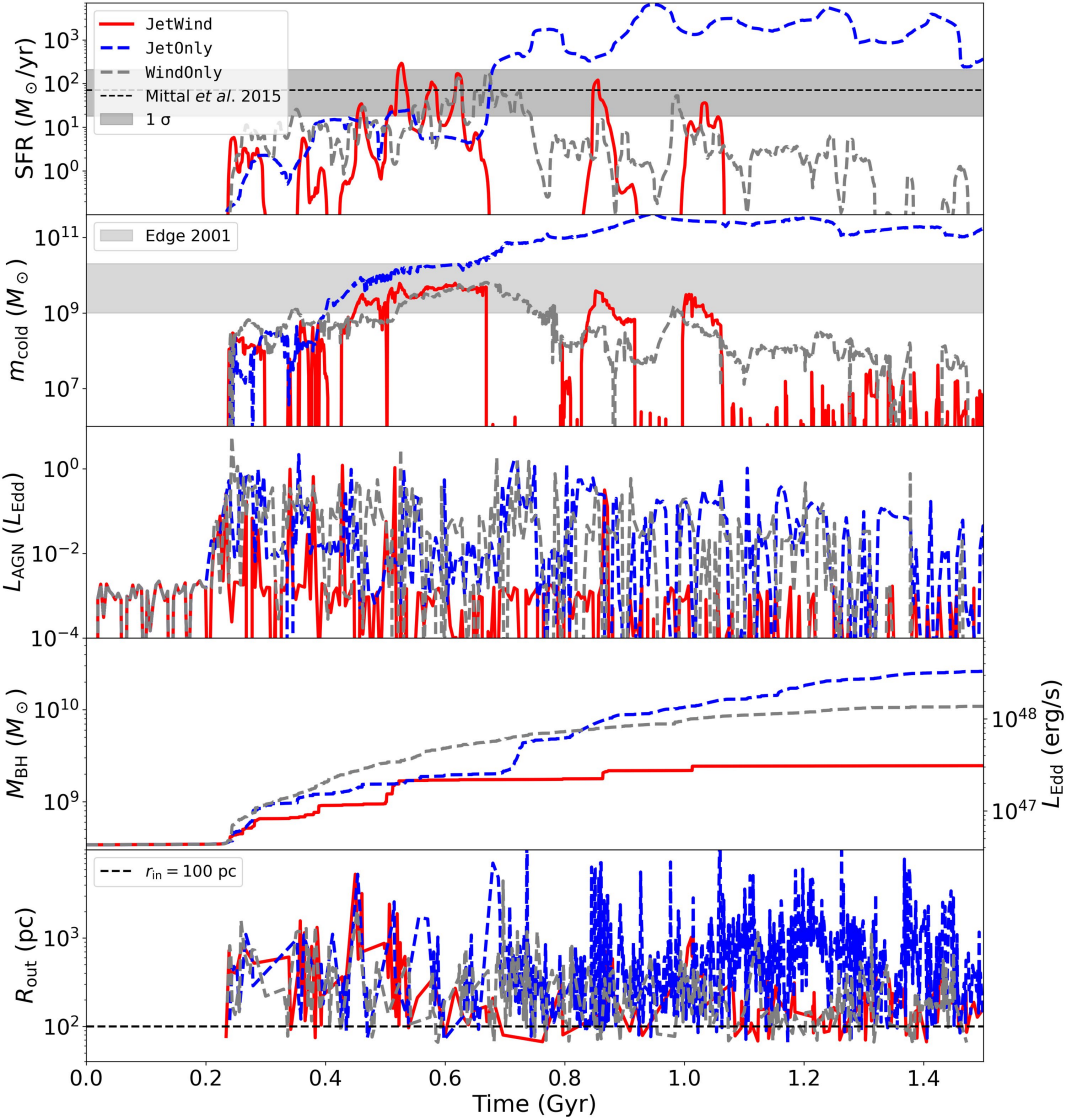
Time evolution of key quantities in the three models. Results are shown for the JetWind (solid red), JetOnly (dashed blue), and WindOnly (dashed gray) models. Top: SFR. The black dashed line and gray-shaded region mark the SFR of NGC 1275 (the Perseus BCG) and its 68% confidence interval (*40*), respectively. yr, year. Second: Cold gas mass. The gray band denotes the molecular gas mass observed in Perseus (*41*). The observational values for SFR and cold gas mass represent upper limits for Perseus-like clusters. Third: AGN bolometric luminosity normalized by the Eddington luminosity. Fourth: Black hole mass and Eddington luminosities. Bottom: Evolution of the mass flux–weighted outer boundary of the accretion flow $R_{\text{out}}$.

Compared with previous idealized cooling flow simulations and cosmological simulations, the MACER framework has several distinctive features. A detailed comparison is presented in Materials and Methods; here, we briefly summarize the most salient ones. First, the inner boundary of our simulation domain is smaller than the outer boundary of the black hole accretion flow, whose value is shown in the bottom panel of Fig. 2. In this case, by combining the mass flux calculated at the inner boundary with black hole accretion theory, we can obtain a reliable estimate of the mass accretion rate at the black hole horizon. This is crucial for studying AGN feedback, given that the accretion rate determines the power of each component of AGN outputs, including the jet.

Second, state-of-the-art accretion physics is adopted in MACER. The black hole accretion is divided into hot and cold modes depending on whether the accretion rate is below or above 2% of the Eddington rate. This boundary value is based on observations of the state transition in black hole x-ray binaries (*28*). In each mode, we consider all AGN outputs, namely radiation, jets, and winds. Specifically, in the hot accretion mode, jet and wind are incorporated simultaneously. Moreover, the parameter values of these outputs as functions of accretion rate, such as the opening angle, velocity, mass flux, and their angular distributions, are not treated as free parameters. Instead, they are directly adopted from, or extrapolated on the basis of, theoretical and observational studies on black hole accretion [(*28*, *29*); see Materials and Methods for details]. This contrasts with other simulation works and ensures that the winds and jets adopted in our model are physically motivated. As we will show later, the coexistence of wind and jet plays an important role in our feedback model, as the coupling between them produces strong turbulence whose dissipation efficiently converts AGN kinetic energy into the thermal energy of the ICM in the galaxy cluster.

### Simulation results and comparison with observations

To assess the importance of including both wind and jet, we conduct three simulations that consider different AGN components in the hot mode: JetWind, JetOnly, and WindOnly. The JetWind simulation includes both jet and wind, while JetOnly and WindOnly disable the wind and jet, respectively. When comparing JetOnly and WindOnly with JetWind, the total outflow power at the same mass accretion rate is kept identical. For instance, in the WindOnly simulation, the jet power is redistributed into the wind channel, ensuring that the removal of one component does not reduce the total feedback power.

During the first 200 million years (Myr), there is almost no cold gas or star formation, and correspondingly, the AGN is very weak. This arises because only hot gas is included in the initial conditions of our simulations. After ∼200 Myr, a cooling flow develops, leading to the formation of cold gas through radiative cooling and a corresponding rise in the SFR. The infall of cold gas onto the black hole triggers strong AGN activity and feedback in all three models.

The feedback effects differ among the three models, as shown in Fig. 2. The JetOnly and WindOnly simulations are less effective than JetWind in suppressing cold gas and star formation. The JetWind model yields the lowest cold gas mass and SFR, followed by WindOnly and then JetOnly. The cold gas mass and SFR in both the JetWind and WindOnly models are consistent with observations, whereas the JetOnly model overpredicts them, producing too much cold gas and too high SFR during most of the evolution. However, if we consider the statistical results of many Perseus-like cooling flow sources, the WindOnly model would fall toward the extreme cases in that sample (*34*).

While AGNs in most galaxy cluster centers are in the hot mode, observations show that a small fraction appears to be in the cold mode (i.e., quasars). The evolution of AGN luminosity shown in Fig. 2 is consistent with this observational result.

Although the SFR and cold gas mass in the WindOnly model are consistent with observations, Fig. 2 shows that the black hole mass in this model (and in the JetOnly model) grows to nearly (and above) $10^{10}\;M_{⊙}$ over the 1.5-Gyr evolution. Such values substantially exceed the observed black hole mass for the Perseus cluster, which ranges from $3.4\times 10^{8}\;M_{⊙}$ (*35*) to $1.1\;\text{to}\;1.2\times 10^{9}\;M_{⊙}$ (*36*). The excessive black hole growth in the WindOnly and JetOnly models arises from inefficient feedback, allowing too much cold gas to form and accrete. In contrast, in the JetWind simulation, the black hole mass remains at a reasonable value throughout the evolution, indicating a more effective feedback regulation. We will discuss the physical reasons for this later.

**Fig. 3.**
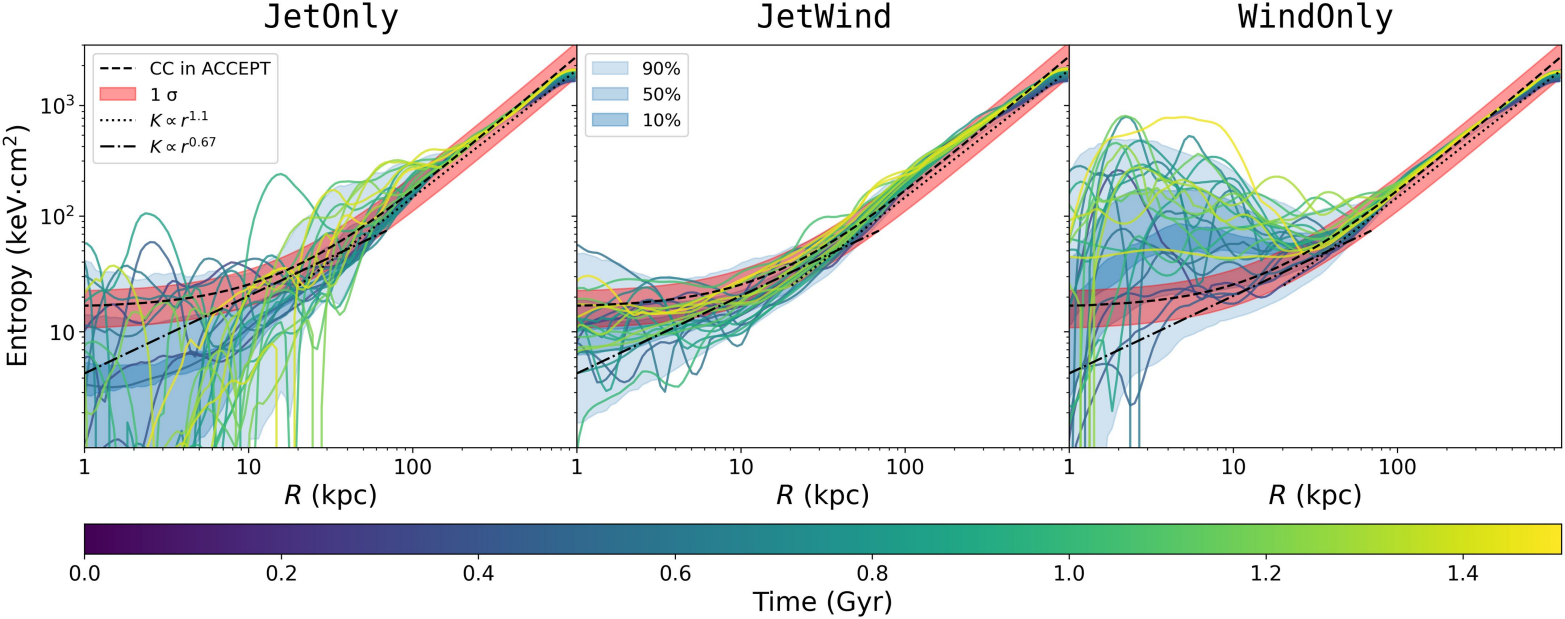
Radial profiles of the entropy of the ICM, color coded by time. The shaded regions denote the 10th to 90th percentile distribution of the simulated profiles. Dashed black lines and red bands indicate the median and 1σ observed profiles from the ACCEPT cluster sample, respectively (*37*). The dot-dashed and dotted lines represent the $K\propto r^{0.67}$ and $K\propto r^{1.1}$ power-law fits, respectively (*38*). Notably, only the JetWind model reproduces observed entropy profiles throughout most of its evolution.

In addition to cold gas mass, SFR, and black hole mass, another important observational constraint is the radial profile of the gas thermodynamic quantities. Figure 3 shows the radial profiles of density-weighted entropy by the three models, along with observational data for comparison (*37*, *38*). The radial profiles of density and temperature with observational data for three models are plotted in fig. S2. The curves are color coded to represent simulation time. For the JetOnly model, within ∼100 kpc, the entropy and temperature are clearly below the observed values, while the density is higher than observed during most of the simulation, indicating that the jet alone fails to adequately heat the ICM in the cluster core. Between 100 and 200 kpc, at late stages of the simulation, moderate increases in both entropy and temperature are observed, suggesting that feedback can affect the cluster outskirts (*39*).

For the WindOnly model, during most of the simulation time, the predicted entropy is substantially higher than observed (first row) because of overly high temperatures and low densities (see fig. S2). Unlike the JetOnly case, regions beyond 100 kpc remain almost unaffected, indicating that wind feedback primarily influences the central ICM regions. This occurs because the wind’s opening angle is much larger than that of the jet and its power is smaller, so the wind’s energy is deposited closer to the AGN.

Figure 2 shows the time evolution of several key quantities in the three models, including the SFR, cold gas mass, AGN bolometric luminosity, black hole mass ($M_{\text{BH}}$) and its corresponding Eddington luminosities ($L_{\text{Edd}}$), and outer boundary of the accretion flow ($R_{\text{out}}$). In the multiphase ICM environment, each phase has its corresponding $R_{\text{out}}$. The outer boundary shown in the figure is the mass flux–weighted value of different phases. The black dashed line represents the observed SFR in NGC 1275 (the BCG in Perseus) measured in (*40*), with the 68% plausible interval indicated by the gray-shaded region. Additional constraints on the cold gas mass from (*41*) are also shown. These observational measurements are “snapshot” estimates for this source and should be regarded as upper limits, given that the statistical averages for cooling flow systems are typically lower (*40*).

In contrast, the JetWind model reproduces the correct radial profiles of entropy, density, and temperature across the entire simulation domain, consistent with observations of cool-core clusters (*37*, *38*). Combined with the results in Fig. 2, we conclude that the JetWind model’s predictions are consistent with all key observations, successfully resolving the cooling problem in galaxy clusters.

**Fig. 4.**
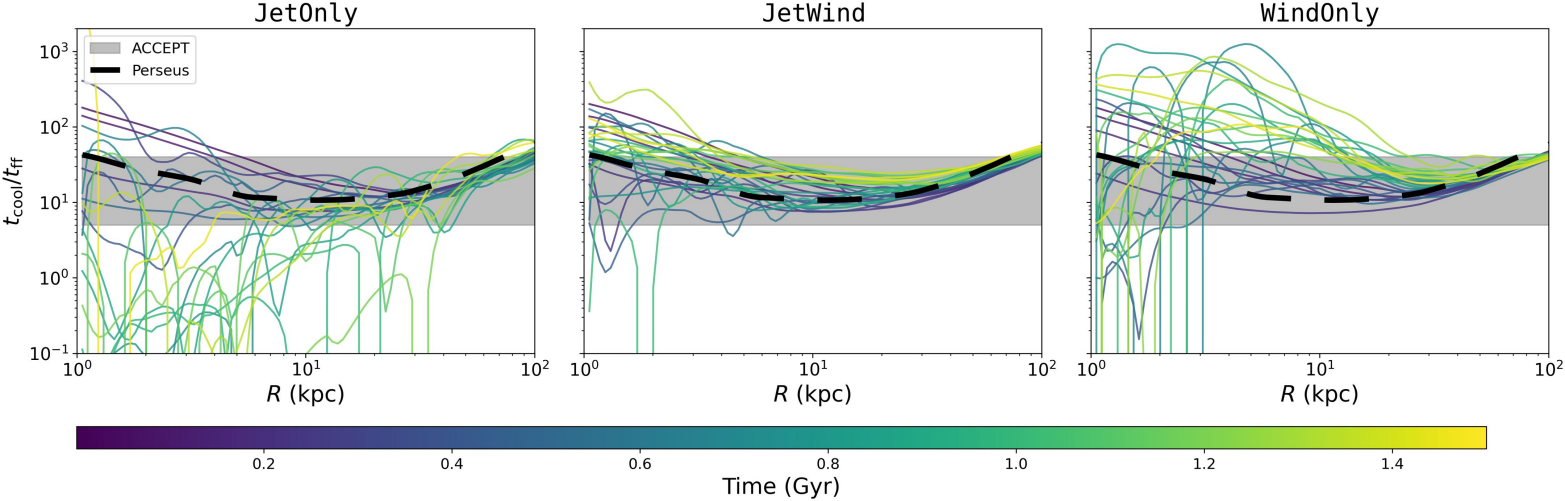
Radial profiles of the density-weighted cooling–to–free-fall time ratio. Colored curves indicate simulation times, as shown by the color bar. The gray-shaded regions mark observational ranges for cool-core clusters from the ACCEPT database (*44*), while the black dashed line shows Perseus data (*41*, *77*). Only the JetWind model maintains $t_{\text{cool}}/t_{\text{ff}}$ values, consistent with observations across the core region.

We further examine two additional diagnostics. The first one is the ratio of the cooling time to the free-fall time. Many analytical and simulation studies have shown that when the minimum value of $t_{\text{cool}}/t_{\text{ff}}$ falls below a certain threshold $t_{\text{cool}}/t_{\text{ff}}<10$ (*10*, *42*, *43*), thermal instability is triggered, leading to the formation of large amounts of cold gas. Figure 4 shows the radial profiles of $t_{\text{cool}}/t_{\text{ff}}$, weighted by density, predicted by the three models. The gray-shaded regions represent observed values from the core regions (within 100 kpc) of cool-core galaxy clusters in the ACCEPT database (*44*). Only the JetWind model agrees with the observations, while the JetOnly and WindOnly models predict ratios that lie below and above the observed range, respectively.

We also analyze the turbulence properties predicted by the three models. In the JetWind model, the velocity power spectrum within 30 kpc closely reproduces the slope and shape of the short-wavelength portion of the Hα velocity structure function observed in (*45*), while the average velocity dispersion of 150 to 200 km/s quantitatively matches the x-ray observations in (*46*). The JetOnly and WindOnly models fail to produce results consistent with observations.

### Interpretation: Why only the jet-wind model can solve the cooling flow problem

Whether the cooling flow problem can be resolved depends on the competition between heating and cooling in the ICM. To understand why only the JetWind model succeeds, we first examine the energetics. Although the AGN power in the JetWind model is the lowest among the three models, its cooling is also the weakest, leading to the smallest amount of cold gas (Fig. 2). We have calculated the total AGN energy and the cooling energy of the ICM released during the whole evolution for the three models. The values are $1.9\times 10^{62}$ and $1.8\times 10^{62}$ erg for JetWind, $4.2\times 10^{62}$ and $6.6\times 10^{62}$ erg for WindOnly, and $1.4\times 10^{63}$ and $2.9\times 10^{63}$ erg for JetOnly, respectively. So, among the three models, only in JetWind does the time-integrated AGN power exceed the cumulative ICM cooling loss.

This outcome arises from the self-regulated nature of the system. A higher ratio of total cooling to heating energy integrated over some period leads to more cold gas, which in turn enhances accretion and AGN heating, thereby reducing the cooling-to-heating ratio. In such a feedback loop, the key determinant of the cold gas content is the efficiency of converting AGN power into the thermal energy of the ICM. A higher conversion efficiency yields less cold gas for a given accretion rate, helping to suppress the cooling flow. We therefore expect JetWind to have the highest efficiency among the three models.

To test this, we computed the ratio of the time-integrated heating energy to the AGN energy released through jets and winds (excluding radiation). The radiative heating rate, given by $\propto n^{2}\left(T_{C}-T\right)F$, with n being the number density, T being the temperature of the gas, $T_{C}$ being the Compton temperature of the AGN radiation, and F being the radiation flux, is most effective at small radii where both n and F are high but is insufficient to offset cooling at larger radii (*17*, *47*). A total of 85% of the radiative heating occurs within 1 kpc. By contrast, jet and wind energy is dissipated mainly through turbulence and shocks at larger scales.

**Fig. 5.**
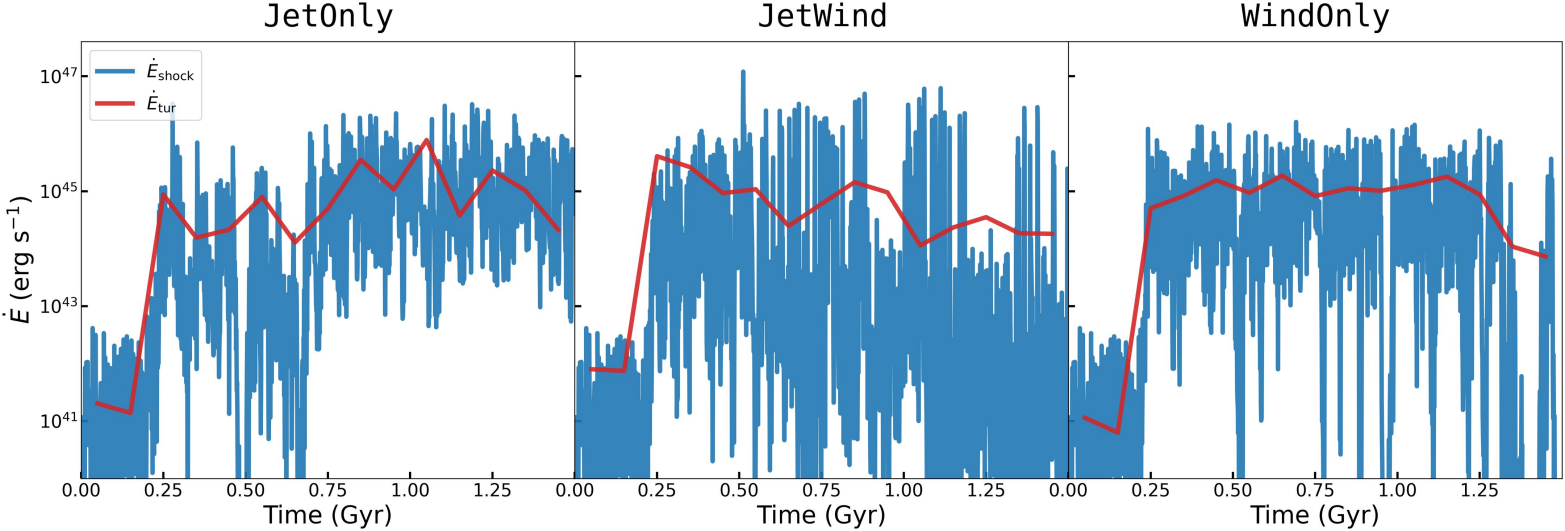
Time evolution of turbulence dissipation rate and shock heating rate.

The calculation of turbulent dissipation rate and shock heating rate is presented in Materials and Methods. Figure 5 shows the time evolution of turbulence dissipation rate and shock heating rate for the three models. The time-integrated turbulence dissipation energies for JetWind, WindOnly, and JetOnly are $E_{\text{tur},\text{total}}=4.6\times 10^{61},3.9\times 10^{61},\;\text{and}\;6.0\times 10^{61}$ erg, respectively. The converting efficiencies, defined as the fraction of the total released kinetic AGN energy that is dissipated by turbulent dissipation, are 29.6,9.3, and 5.2%, respectively. The respective shock-heating energies for the three models are $E_{\text{shock},\text{total}}\approx 5.1\times 10^{61},3.6\times 10^{61},\;\text{and}\;5.7\times 10^{61}$ erg, respectively, with the shock energy conversion efficiencies of 33.0,8.6, and 4.9%, respectively. Combining the turbulent dissipation and shock heating yields total conversion efficiencies of 62.6,17.9, and 10.1%. Thus, JetWind exhibits the highest efficiency, confirming our expectation and explaining why it alone eliminates the cooling flow.

A deeper question is why JetWind achieves such high efficiency. In our simulations, turbulence is mainly produced by the shear between jet, wind, and ambient ICM through the Kelvin-Helmholtz instability, cascading to smaller scales and converting kinetic to thermal energy. The turbulence amplitude in JetWind is the largest (see Materials and Methods), accounting for its superior energy-conversion efficiency.

## DISCUSSION

Our simulations presented in this work are idealized and do not account for cosmological processes such as mergers and large-scale cosmological inflows. We note that most of clusters are actively accreting and have experienced recent merger events; therefore, caution is required when comparing our simulation results with observations. Nevertheless, some studies have suggested that mergers, cosmological inflows, and substructure evolution play only a minor role in suppressing cooling flows (*48*, *49*). In future work, we plan to systematically investigate these effects.

AGN feedback plays a fundamental role not only in alleviating the cooling flow problem but also in regulating galaxy formation and evolution more broadly. Jets constitute a primary channel for energy injection in AGN feedback, underscoring the importance of understanding how their energy is deposited into the host galaxy. Our results suggest that the coupling between jets and winds can provide an efficient mechanism for converting AGN kinetic power into effective feedback on the host system, particularly in massive halos where sustained AGN activity is present. This process therefore merits explicit consideration in future theoretical and numerical studies depending on the physical regime and scientific goals being addressed.

## MATERIALS AND METHODS

### Introduction to the MACER3D framework

The simulations were carried out using MACER3D, a comprehensive 3D extension of the earlier 2D MACER framework. The MACER project builds upon a series of foundational studies on the coevolution of supermassive black holes (SMBHs) and their host galaxies (*50*, *51*). The most recent 2D MACER framework (*33*) incorporates state-of-the-art prescriptions for AGN radiation and winds as functions of accretion rate, including radiative heating and cooling processes such as bremsstrahlung, Compton heating/cooling, photoionization heating, and line and recombination continuum cooling. Additional physical processes, such as star formation and stellar feedback, are also included. While the MACER framework technically supports the inclusion of cosmological inflow (*52*), this feature was explicitly disabled in this study. The inner boundary of the simulation domain is set smaller than the outer boundary of the black hole accretion flow. This setup allows the mass flux measured at the inner boundary to be reliably combined with black hole accretion theory to determine the precise mass accretion rate at the event horizon, which is the key parameter governing AGN power and feedback strength. MACER3D (*32*) further extends this framework by introducing physically motivated models for supernova feedback, radiative cooling, and metal enrichment. The implementation of the grid-based Athena++ code (*53*), using a low-diffusion HLLC Riemann solver and a second-order Runge-Kutta (RK2) time integrator, enables robust 3D modeling of nonaxisymmetric instabilities and multiphase gas dynamics. Given that no explicit viscosity is incorporated into the simulation setup, the resolved turbulence is effectively determined by the numerical diffusion of the solver (*53*), allowing for the treatment of complex physical features that were inaccessible to earlier 2D axisymmetric versions (*33*). Radiative cooling is calculated via exact integration as described in (*32*).

Our simulations solve the 3D hydrodynamic equations in spherical coordinates (r,θ,ϕ) using the standard Euler form

$$\frac{\partial \rho }{\partial t}+\nabla \cdot \left(\rho v\right)=-{\dot{\rho }}_{⋆}^{+}$$ (1)

$$\frac{\partial m}{\partial t}+\nabla \cdot \left(\text{mv}\right)=-\nabla p_{\text{gas}}+\rho g--\nabla p_{\text{rad}}-{\dot{m}}_{⋆}^{+}$$ (2)

$$\frac{\partial E}{\partial t}+\nabla \cdot \left(Ev\right)=-p_{\text{gas}}\nabla \cdot v+H-C-{\dot{E}}_{⋆}^{+}$$ (3)

where ρ, m, and E are the gas mass, momentum, and internal energy per unit volume; v and g are the velocity and the gravitational field of the galaxy cluster; and ${\dot{\rho }}_{⋆}^{+}$, ${\dot{m}}_{⋆}^{+}$, and ${\dot{E}}_{⋆}^{+}$ denote the sink terms of mass, momentum, and energy due to star formation, respectively. The gas pressure is $p_{\text{gas}}=\left(\gamma -1\right)E$ with adiabatic index γ=5/3, and $\nabla p_{\text{rad}}$ represents the radiation force (*33*). Last, H and C denote the radiative heating and cooling rates, respectively. The computational domain extends from an inner boundary of $r_{\text{in}}=100$ pc to an outer boundary of $r_{\text{out}}=2$ Mpc, a configuration designed to simultaneously resolve gas accretion flows near the SMBH and large-scale environmental effects within the galactic halo. We use a fiducial resolution of 256×64×128 cells. While the angular coordinates (θ,ϕ) are uniformly discretized, the radial grid is logarithmically spaced with a constant scaling ratio $\Delta r_{i+1}/\Delta r_{i}=1.038$. This grid configuration enhances the resolution in the cluster core, achieving a peak spatial resolution of ∼5 pc at the inner boundary.

In the MACER framework, black hole accretion operates in two distinct modes, namely hot and cold modes, separated by 2% $L_{\text{Edd}}$ (*28*). In the cold mode, gas infalling through the inner boundary forms a thin disc at the circularization radius. The disc is continuously fed by the inflow, while mass is depleted through accretion onto the black hole and through winds launched from the disc surface. Solving the corresponding set of differential equations yields the black hole mass accretion rate ${\dot{M}}_{\text{BH}}$ (*33*). In this mode, the mass flux and velocity of the wind are determined from the observed statistical relation between AGN luminosity and outflow properties (*33*). The cold wind mass flux and velocity are

$${\dot{M}}_{W,C}=0.28{\left(\frac{L_{\text{bol}}}{10^{45}\;\text{erg}\;s^{-1}}\right)}^{0.85}\;M_{⊙}\;{\text{year}}^{-1}$$ (4)

$$v_{W,C}=2.5\times 10^{4}{\left(\frac{L_{\text{bol}}}{10^{45}\;\text{erg}\;s^{-1}}\right)}^{0.4}\;\text{km}\;s^{-1}$$ (5)

where $L_{\text{bol}}=0.1\;{\dot{M}}_{\text{BH}}c^{2}$ is the bolometric luminosity. The mass flux of the wind adopts a bipolar angular distribution ∝θ with a half-opening angle $\theta_{W,C}=60{}^{\circ}$ at the inner boundary.

Hot accretion flows have been extensively studied (*28*). In the hot mode, the accretion flow consists of an outer truncated thin disc and an inner hot accretion flow, with the truncation radius $r_{\text{tr}}=3r_{s}\left[\frac{2\times 10^{-2}{\dot{M}}_{\text{Edd}}}{\dot{M}\left(r_{\text{in}}\right)}\right]$ determined solely by the accretion rate of the thin disk. Magnetohydrodynamic simulations demonstrate that powerful winds are launched across the entire hot accretion region (*26*–*29*), a prediction increasingly supported by observations (*30*, *54*–*56*). Following the hot accretion flow theory (*29*, *33*), given the mass inflow rate $\dot{M}\left(r_{\text{in}}\right)$ at the inner boundary, the black hole accretion rate can be calculated in (*29*)

$${\dot{M}}_{\text{BH}}=\dot{M}\left(r_{\text{in}}\right)\;{\left(\frac{2.5r_{s}}{r_{\text{tr}}}\right)}^{0.42}$$ (6)

Given the limited observational constraints of hot wind, MACER adopts wind properties derived from 3D general relativistic magnetohydrodynamic (GRMHD) simulations (*29*). Specifically, the mass flux and velocity of hot wind are

$${\dot{M}}_{W,H}=\dot{M}\left(r_{\text{in}}\right)\;\left[1-{\left(\frac{2.5r_{s}}{r_{\text{tr}}}\right)}^{0.42}\right]$$ (7)

$$v_{W,H}=0.64\;v_{k}\left(r_{\text{tr}}\right)$$ (8)

where $\dot{M}\left(r_{\text{in}}\right)$ is the inflow mass flux at the inner boundary $r_{\text{in}}=100\;\text{pc}$, and $r_{s}=2GM_{\text{BH}}/c^{2}$ is the Schwarzschild radius. The wind velocity is determined by the Keplerian speed at the truncation radius $v_{k}\left(r_{\text{tr}}\right)$ where most of the wind mass flux originates, beyond which the poloidal velocity of wind remains approximately constant (*33*). The angular distribution of the hot wind is restricted to 10° to 50° and 130° to 170° (*29*). The black hole accretion rate at the event horizon is then computed by combining the measured mass flux at the inner boundary of the simulation domain and truncation radius with the theory of wind from hot accretion flows (*33*).

The AGN releases radiation, winds, and jets. Previous MACER studies included only radiation and winds while neglecting jets, which play a crucial role in addressing the cooling flow problem. In the next section, we describe in detail how jets are implemented in the present work. Once the properties of all AGN outputs are determined, they are injected at the inner boundary of the simulation domain, and their energy and momentum exchanges with the ICM are computed self-consistently. This approach avoids the use of parameterized, phenomenological feedback prescriptions commonly used in earlier simulations.

### Modeling of the AGN jet

In almost all existing subgrid jet feedback models, the key jet parameters are treated as almost unconstrained free variables, whose values are not necessarily consistent with the constraints obtained from GRMHD simulations of black hole accretion and jet formation (*29*, *57*–*59*). The formation and properties of jets are largely determined by two factors: the black hole spin and the accretion mode [standard and normal evolution (SANE) or magnetically arrested disk (MAD)]. Current studies suggest that powerful jets are launched in the MAD mode (*60*, *61*). We adopt a high dimensionless spin parameter, *a* = 0.98, consistent with spectral and timing constraints on massive, cluster-center AGN (*62*, *63*). Under these assumptions, the jet parameters are taken from the 3D GRMHD simulations of MAD accretion onto rapidly spinning black holes presented in (*29*). In that work, the jet (and wind) properties were derived using the “virtual test-particle trajectory” approach, which can more accurately separate turbulent motion from genuine outflow compared to the commonly used “streamline” method, thus providing more reliable jet (and wind) parameters. Given that the outer boundary of the simulations in (*29*) extends only to $∼10^{3}\;r_{g}$ (where $r_{g}=GM_{\text{BH}}/c^{2}∼1.67\times 10^{-5}$ pc is the gravitational radius), much smaller than the inner boundary of our simulation domain ($100\;\text{pc}∼6\times 10^{6}\;r_{g}$), extrapolation of the GRMHD results is required for implementation in MACER.

The first quantity to determine is the total jet energy flux. GRMHD numerical simulations and analytical studies show that after launch, the Poynting flux of the jet is efficiently converted into kinetic and thermal energy within the acceleration zone (up to parsec scales) through magnetohydrodynamic processes and internal shocks (*57*, *64*). We assume that the total jet energy flux remains conserved during this process and adopt the value given in (*29*)

$${\dot{E}}_{\text{jet}}={\dot{E}}_{\text{kin},\text{jet}}+{\dot{E}}_{\text{th},\text{jet}}=0.9{\dot{M}}_{\text{BH}}c^{2}$$ (9)

where ${\dot{E}}_{\text{kin},\text{jet}}$ and ${\dot{E}}_{\text{th},\text{jet}}$ denote the kinetic and thermal components, respectively.

The second key parameter is the jet velocity. The mass flux–weighted jet velocity at $200\;r_{g}$ ($∼3.3\times 10^{-3}$ pc) obtained in (*29*) is 0.5 c. Given that the inner boundary of our MACER simulations is much larger than the outer boundary in (*29*) and other typical GRMHD simulations of jet formations, we adopt the observed jet speed as the injection jet speed in our model. Although there is no direct measurement of the jet speed at ∼100 pc, observational constraints are fairly tight. High-resolution millimeter very-long-baseline interferometry observations of the jet in the Perseus cluster reveal an apparent speed of 0.055 to 0.22 c at the ∼1-pc scale (*65*). The jet viewing angle in this source is estimated to be 10° to 35°. Furthermore, a detailed kinetic study in (*66*) indicates that the jet is subrelativistic and maintains an approximately constant speed from ∼1 pc to many parsecs, showing no sign of acceleration. The jet in the Perseus cluster is a relatively weak FR I jet that propagates through the compact core of the Perseus cluster. Combining the above information, we adopt the jet velocity at the inner boundary of our simulation domain to be

$$v_{\text{jet}}=0.1\;c$$ (10)

Given that we assume that the sum of kinetic power and the thermal power of the jet remains unchanged, we increase the thermal energy of the jet to satisfy this condition.

The third key parameter is the jet mass flux. To estimate this quantity, we have reanalyzed the original GRMHD simulation data presented in (*29*) and derived the mass flux of the jet at a distance of $1000\;r_{g}$ ($∼3.3\times 10^{-3}$ pc)

$${\dot{M}}_{\text{jet}}=0.35\;{\dot{M}}_{\text{BH}}$$ (11)

We assume that the mass flux remains roughly unchanged out to $6\times 10^{6}\;r_{g}$ (100 pc) (*67*).

The fourth key parameter is the jet half-opening angle $\theta_{j}\equiv \left(R/Z\right)$, where R and Z are the half-radius and the distance from the black hole, respectively. There is no direct observational constraint on the value of this angle, so we use the theoretical result. Reference (*29*) found that the jet radius follows a power-law relation with distance, $R=1.01\;Z^{0.8}$. Using this profile, we calculate the half-opening angle of the jet at $∼10^{4}\;r_{g}$ to be ∼7.5°. Beyond this radius, up to $10^{7}\;r_{g},$ observations indicate that the opening angle of the jet roughly remains constant (*68*). Therefore, we adopt a half-opening angle of the jet at the inner boundary of our simulation domain to be

$$\theta_{j}\approx 7.5{}^{\circ}$$ (12)

This value is roughly consistent with the observational result reported in (*67*). The jet is initialized in the ghost zones at each time step using its prescribed mass flux and velocity, with its thermal power incorporated as the internal energy, and is subsequently launched from the injection regions at the inner boundary.

Last, although ref. (*29*) found some angular dependence of velocity within the jet, ref. (*68*) showed that this variation becomes increasingly flattened as the jet propagates outward. Accordingly, in the present work, we assume a uniform velocity across the jet cross section. No ad hoc jet precession is imposed.

### Comparison between MACER and other models

In this section, we compare the MACER model with other AGN feedback models, including both idealized cooling flow simulations and large-scale cosmological simulations.

1) Accretion and feedback modes: According to the black hole accretion theory, accretion proceeds in two distinct modes, namely hot and cold modes, depending on the normalized black hole accretion rate. These two accretion modes naturally correspond to two feedback modes. Jet exists only in the hot mode, while winds are expected to exist in both modes (*28*). The MACER model explicitly incorporates this two-mode accretion-feedback paradigm: In the hot mode, both jets and winds are self-consistently included, while in the cold mode, only winds are present.

2) Many existing idealized cooling flow simulations do not adopt this two-mode framework and instead implement only jet feedback [e.g., (*10*–*19*)].

3) Cosmological simulations exhibit substantial diversity in their treatment of AGN feedback. EAGLE does not distinguish between hot and cold accretion modes and implements AGN feedback exclusively through isotropic thermal energy injection without explicitly modeling jets or winds (*69*). IllustrisTNG adopts a two-mode feedback scheme, but neither mode explicitly models jets or winds; instead, feedback is implemented phenomenologically (*70*). COLIBRE does not enforce a strict two-mode dichotomy. It includes both jet and wind feedback but allows both components to operate simultaneously across different accretion states (*71*). SIMBA implements a two-mode framework, in which winds operate in the cold mode and jets are launched exclusively in the hot mode, but the two components do not coexist (*72*). Among current cosmological simulations, OBSIDIAN adopts a feedback structure most similar to MACER, allowing both jets and winds to coexist in the hot mode while including only winds in the cold mode (*73*).

4) Jet opening angle: In current cosmological simulations, AGN jets are generally implemented using subgrid or phenomenological prescriptions, and their physical properties vary substantially among different models. In IllustrisTNG, collimated relativistic jets are not explicitly modeled; instead, the low-accretion-rate feedback mode is implemented as kinetic energy injection with weak or no explicit collimation and without well-defined jet velocity, opening angle, or mass flux. SIMBA, COLIBRE, and OBSIDIAN include more directional outflows that are sometimes referred to as jets; however, their velocities, mass-loading factors, and opening angles are typically fixed or semiempirical parameters chosen to reproduce galaxy-scale observables, rather than being derived from black hole accretion physics. For this reason, the following comparisons of jet properties focus primarily on idealized cooling flow simulations.

5) In MACER, we adopt a jet half-opening angle of 7.5°, a value motivated by 3D GRMHD simulations of black hole accretion and jet formation. The jet is assumed to be nonprecessing. In other works, jets are usually modeled as bipolar precessing outflow, with assumed precessing angle $\theta_{\text{prec}}$ ranging from 8.6° (*13*, *17*) to 15° (*14*), 25° (*10*, *19*), and 30° (*18*), typically with an assumed precession period of ∼10 Myr. In these models, the outflow is injected along the *z* axis from two parallel “jet launching planes” located at a height $h_{j}$ from the black hole, each with a radius $R_{j}$. This setup corresponds to an effective jet half-opening angle of $\text{arctan}\left(R_{j}/h_{j}\right)\approx \theta_{j}=30{}^{\circ}$ (*12*), 37° (*13*), 45° (*17*), 51° (*14*), and 66° (*18*). These values are substantially larger than those inferred from observations or GRMHD numerical simulations of jet formation. Moreover, the injected jet velocity in these models is purely along the *z* direction, unlike the radially oriented velocity field expected for physically launched jets. Although some of these studies adopt relatively large jet opening angles that may partially resemble a combined wind-jet outflow, other jet properties, such as velocity, density, and their angular dependence, remain fundamentally different from the physically motivated wind + jet properties implemented in MACER.

6) Jet mass flux: In MACER, the jet mass flux is prescribed as a function of the black hole accretion rate on the basis of the 3D GRMHD simulations of black hole accretion. In contrast, many other works treat the jet mass flux as a free parameter, often without a clear physical justification for the adopted value. As a result, the assumed jet mass-loading factors vary widely, ranging from ${\dot{M}}_{\text{jet}}/{\dot{M}}_{\text{BH}}∼1$ (*13*, *14*, *17*, *18*) to ∼0.432 (*10*, *19*) and down to ∼0.006 (*12*).

7) Jet velocity: In our work, the jet velocity (0.1 c) is constrained by a combination of radio observations of the jet in the Perseus cluster and results from 3D GRMHD simulations of black hole accretion, as we have explained in the last section. In previous studies (*10*–*14*, *17*–*19*), the adopted jet velocities span a wide range, from 0.16 c (*10*, *19*) and 0.1 c (*12*, *13*), down to 0.045 c (*14*, *17*) and 0.033 c (*18*). Our adopted jet velocity therefore lies well within the range assumed in previous works.

8) Wind launched from the black hole accretion flow. In MACER, the properties of wind, including the opening angle, velocity, density, and their angular and accretion-rate dependences, are largely taken from GRMHD simulations of black hole accretion in the hot mode and observations in the cold mode. These properties differ substantially from those of the jet. In particular, the angular distribution of outflow velocity plays a crucial role. As demonstrated in the present work, a physically consistent combination of wind and jet velocities naturally generates strong turbulence, whose dissipation efficiently converts AGN kinetic power into the thermal energy of the ICM.

9) As noted above, most idealized cooling flow simulations do not include winds. Among cosmological simulations, EAGLE does not explicitly model winds or jets, and it implements AGN feedback exclusively through isotropic thermal energy injection (*69*). IllustrisTNG also does not explicitly model winds or jets. The hot mode uses kinetic energy injection, but this kinetic feedback is implemented phenomenologically without explicitly modeling collimated jets or physically motivated AGN winds launched from the accretion flow. In the cold mode, there is no wind either, and the AGN feedback is implemented in a way similar to EAGLE (*70*). Although COLIBRE includes a component referred to as “wind,” its implementation is fundamentally different from the physically motivated accretion-driven winds in MACER. In COLIBRE, the wind is implemented phenomenologically as part of the AGN kinetic feedback, whereas in MACER, the wind properties are directly constrained by black hole accretion theory and GRMHD simulations (*71*). SIMBA launches winds in the cold mode, but the wind is implemented phenomenologically, with its properties calibrated to observations rather than derived from black hole accretion physics (*72*). Although OBSIDIAN is the most physically comparable to MACER, the wind and jet properties are not directly constrained by GRMHD simulations but are implemented using phenomenological subgrid prescriptions tied to the accretion rate and feedback efficiency (*73*).

10) Black hole accretion rate: In MACER, the value of the inner boundary of the simulation domain is smaller than the outer boundary of the accretion flow (see Fig. 2). This enables a physically motivated calculation of the accretion rate at the black hole horizon by combining the mass flux at the inner boundary with black hole accretion theory [see (*33*) for details].

11) In contrast, in most other works, the accretion rate is only crudely estimated. In idealized cooling flow simulations, it is often calculated by dividing the total amount of cold gas within an arbitrarily assumed radius by an assumed accretion timescale, with little justification for the adopted radius (*12*–*14*, *17*–*19*)*.* In cosmological simulations, the Bondi or modified Bondi accretion rate is commonly applied in both hot and cold accretion modes to estimate the black hole accretion rate (*69*–*71*, *73*) or hot mode for SIMBA (*72*). For the cold mode accretion, SIMBA models the gas inflow rate driven by disc gravitational instabilities, while OBSIDIAN considered cold gas accretion using a way similar to idealized cooling flow simulations. We emphasize that if the Bondi radius is unresolved in the simulation, it is not correct to apply the Bondi or modified Bondi formula; otherwise, the errors can be as large as 100 or even much higher [e.g., (*74*, *75*)].

12) Interaction between AGN outflow and ICM/ISM (interstellar medium): In MACER, both wind and jet are injected at the inner boundary, and their momentum and energy interaction with the ICM/ISM is then self-consistently calculated. In contrast, in the “thermal feedback mode” adopted in many cosmological simulations, AGN feedback is typically implemented in a phenomenological manner, namely as isotropic heating of gas within a certain distance from the black hole (*69*–*71*). In such implementations, both the fraction of AGN energy deposited into the gas and the deposition radius are free parameters with limited physical motivation. The momentum feedback effect associated with AGN winds, which may play a more important role than pure energy feedback (*75*), is commonly neglected. Furthermore, to mitigate numerical overcooling, feedback energy is commonly stored in a reservoir until a sufficient amount is accumulated, an approach that again lacks a clear physical basis.

13) Resolution: The highest resolution of MACER is achieved at the small radii, which is ∼5 pc. The highest resolutions of other idealized isolated cluster simulations are ∼15 to 20 pc (*12*), 15 to 60 pc (*13*), 30 to 160 pc (*16*), several hundred parsecs (*17*–*20*), and 1.95 kpc (*14*). In cosmological simulations, spatial resolution is typically constrained by gravitational softening lengths ranging from 0.3 kpc in state-of-the-art runs like TNG50 (*31*) to ∼0.7 kpc in EAGLE (*69*) and SIMBA (*72*).

### Initial conditions of the simulations

We adopt the archetypal, relaxed cool-core galaxy cluster Perseus as a reference system for our simulations while noting that our models are not intended to reproduce this specific cluster in detail because of the lack of cosmological effects such as merger and cosmological inflow in our simulations. Following the work in (*13*), we initialize the ICM as a hydrostatic sphere of gas within a static spherical gravitational potential. The gravitational potential comprises three components: a Navarro-Frenk-White (NFW) halo, the stellar mass profile of the BCG, and the SMBH. The NFW halo density profile is (*76*)

$$\rho \left(r\right)=\frac{\rho_{0}}{\frac{r}{R_{s}}{\left(1+\frac{r}{R_{s}}\right)}^{2}}$$ (13)

where $\rho_{0}$ is found to be $8.42\times 10^{-26}\;g\;cm^{-3}$, and the scale radius $R_{s}=351.7$ kpc. The BCG is treated as a fixed potential (*34*) with the stellar acceleration

$$g_{*}\left(r\right)={\left[{\left(\frac{r^{0.5975}}{3.206\times 10^{-7}}\right)}^{0.9}+{\left(\frac{r^{1.849}}{1.861\times 10^{-6}}\right)}^{0.9}\right]}^{-1/0.9}$$ (14)

The SMBH in the center of the cluster is treated as a point mass of $M_{\text{SMBH}}=3.4\times 10^{8}\;M_{⊙}$ (*35*). Combining the black hole mass and the typical temperature of the hot ICM gas, the inner boundary $r_{\text{in}}$ of our simulations is set to 100 pc, which is roughly equal to the outer boundary of the accretion flow.

We assume an ideal gas law for the ICM with γ=5/3 and that the ICM is initially in hydrostatic equilibrium with the gravitational potential, which includes the contribution from the NFW halo, the BCG, and the SMBH. We do not include any initial rotation or perturbation in the gas. The hydrostatic gas in the halo is initialized with an electron density profile of the form (*39*)

$$n_{e}\left(r\right)=\left\{\frac{0.0192}{1+{\left(\frac{r}{18}\right)}^{3}}+\frac{0.046}{{\left[1+{\left(\frac{r}{57}\right)}^{2}\right]}^{1.8}}+\frac{0.0048}{{\left[1+{\left(\frac{r}{200}\right)}^{2}\right]}^{1.1}}\right\}{\text{cm}}^{-3}$$ (15)

where r is the distance to the cluster center in kpc.

Following (*13*), the initial ICM temperature profile within the central galaxies (r<300 kpc) is constrained by observations (*77*) as

$$T=7\frac{1+{\left(r/71\right)}^{3}}{2.3+{\left(r/71\right)}^{3}}\;\text{keV}$$ (16)

while at larger radii (r>300 kpc), we adopt the universal profile proposed in (*78*)

$$T=9.18\;{\left(1+\frac{3r}{4880}\right)}^{-1.6}\;\text{keV}$$ (17)

Combining with the ICM electron density profile, we have $r_{200}=1.83$ Mpc and $M_{200}=7.47\times 10^{14}\;M_{⊙}$ defined as the radius within which the mean enclosed density is 200 times the critical density. On the basis of this, the outer boundary of our isolated simulations is set to approximately one virial radius as 2 Mpc, which is slightly larger than $r_{200}$ to mitigate the effects of boundary conditions. The power-law index of the last term is slightly steepened so that the density profile at large radii is more consistent with cosmological simulations as well as the observations of the outskirts of Perseus (*35*). Given that we focus on the cluster core within the cooling radius, beyond which both cooling and dynamical timescales are very long, the exact value of the index is not very important. We do not include initial rotation in the gas.

### Three simulation models

In contrast to previous cluster scale simulations that predominantly investigated single-channel feedback mechanisms, MACER3D introduces a framework incorporating a multichannel feedback scheme, including AGN jets as well as hot and cold winds. To systematically assess the importance of properly configuring multichannel feedback in suppressing ICM cooling, we set up three simulations. The JetWind simulation includes both AGN jet and hot wind. For comparison, the JetOnly and WindOnly simulations disable the hot wind (${\dot{M}}_{W,H}=0$) and the jet (${\dot{M}}_{\text{Jet}}=0$), respectively, while keeping other feedback components (e.g., cold wind) active. All three simulations retain the cold wind at high accretion rates. When comparing JetOnly and WindOnly with JetWind, the total feedback power under the same black hole mass accretion rate remains nearly identical; for example, in the WindOnly simulation, we set ${\dot{M}}_{\text{Jet}}=0$ but redistribute the jet power into the hot wind channel, and vice versa. This ensures that removing a specific feedback channel does not reduce the overall AGN output power, thereby allowing a fair comparison of the feedback effects for three simulation sets.
