# Supplementary material for: Solving the cooling flow problem with combined jet-wind AGN feedback
Source: Sci Adv. 2026 Jul 3;12(27):eaed6394. doi: 10.1126/sciadv.aed6394 (PMC13330858; doi:10.1126/sciadv.aed6394)
Supplement: Supplementary file 1 — Supplementary Text Figs. S1 and S2 [file sciadv.aed6394_sm.pdf]

Science Advances
AAAS

Supplementary Materials for

Solving the cooling flow problem with combined jet-wind AGN feedback

Aoyun He *et al.*

Corresponding author: Feng Yuan, fyuan@fudan.edu.cn; Suoqing Ji, sqji@fudan.edu.cn

Sci. Adv. **12**, eaed6394 (2026)
DOI: 10.1126/sciadv.aed6394

This PDF file includes:

Supplementary Text
Figs. S1 and S2

Supplementary Text

Turbulent dissipation rate

Our approach to quantifying this process follows the methodology of (*18*). To avoid the contamination from the radial bulk motions directly injected by the AGN, we compute the velocity power spectrum using the velocity dispersion of v_θ. This choice minimizes the impact of large-scale radial flows and ensures that the derived spectrum more faithfully represents the turbulent velocity field. We define the velocity fluctuation as

$$\delta v(r,\theta,\varphi) = v_\theta(r,\theta,\varphi) - \overline{v_\theta}(r)\,, \tag{S1}$$

where $\overline{v_\theta}(r)$ is the mean velocity at each radius. To avoid the influence of bulk motions in the θ direction caused by the lateral adiabatic expansion of hot bubbles generated by the outflows, the mean velocity and the velocity dispersion are computed separately in the regions with $\theta > \pi/2$ and $\theta < \pi/2$. This separation effectively reduces the contamination from large-scale coherent flows associated with bubble expansion and allows the resulting velocity statistics to better capture the underlying turbulent motions. We excluded the r-direction velocity dispersion because gravity wave damping in stratified media (like the ICM) substantially suppresses radial turbulence at certain positions, making theta-direction dispersion dominant. We also omitted phi-direction dispersion since our simulation is rotationally symmetric without artificial angular momentum.

Over the entire simulation time span of 1.5 Gyr, we obtain the time-averaged values of the velocity amplitude. Next, we perform a Fast Fourier Transform (FFT) of the one-component velocity amplitude to obtain the power spectral density $E_k(k)|_r$ at each fixed radius r, from which the velocity fluctuation field in frequency space can be derived as

$$\delta v(k)|_r = \sqrt{E_k(k)|_r k}, \tag{S2}$$

where for a given radius r, the wavenumber range is $k|_r \in \left(\frac{1}{r\mathrm{d}\theta}, \frac{1}{\pi r}\right)$ with $\mathrm{d}\theta$ denoting the minimum angular resolution in the θ direction. Since the wavenumber spaces corresponding to adjacent radii partially overlap, the resulting complete power spectrum should represent the dominant frequency in each mode. Therefore, we adopt a logarithmic binning scheme over the entire wavenumber range, selecting the maximum power spectral density within each k-bin, from which the corresponding velocity dispersion is then computed. Finally, by combining the velocity power spectra from all radial shells, we construct the complete power spectrum over the full wavenumber range $\delta v(k)$.

Similar to (*18*), we also assume that the turbulent energy density, $\frac{1}{2}\bar{\rho}\delta v(k_{\mathrm{inertial}})^2$, associated with turbulent eddies of size $\frac{1}{2}\bar{\rho}\delta v(k_{\mathrm{inertial}})^3 L \sim 1/k_{\mathrm{inertial}}$, cascades down to smaller eddies on approximately one eddy turnover time, $t_{\mathrm{turn}} = (k\delta v)^{-1}$. Finally, the expression for the turbulent dissipation rate density is given by

$$\dot{e}_{\mathrm{tur}} = \frac{1}{2}\bar{\rho}\delta v(k_{\mathrm{inertial}})^3 k_{\mathrm{inertial}} \tag{S3}$$

where $\bar{\rho}$ denotes the mean density within the spatial region (30 kpc) used to compute the velocity power spectrum, and k_{inertial} is the wavenumber within the inertial range of a fully developed

Kolmogorov turbulence power spectrum. The total turbulent dissipation power is then evaluated as $\dot{E}_{\rm tur} = \dot{e}_{\rm tur}\, V$, where V represents the volume of the spherical region with a radius of 30 kpc.

Theoretically, the turbulent dissipation rate within the inertial range is independent of $k_{\rm inertial}$. To better estimate energy dissipation through energy cascading across different scales, the final turbulent dissipation power in each of the three simulations (`JetWind`, `JetOnly` and `WindOnly`) is obtained by averaging the dissipation rates computed over all wavenumbers within their respective inertial ranges of the power spectrum. This averaging procedure reduces the uncertainty introduced by numerical noise or fluctuations at individual wavenumber modes. This method provides a direct link between the simulated turbulence, energy dissipation, and the resulting heating rate in the ICM.

Shock heating rate

To calculate the shock heating, we adopt an approximate estimator of the shock heating rate, which is applied in post-processing. Our approach follows methods developed in previous works (*14,18*), where shocks are identified through entropy, pressure, and density jumps across consecutive snapshots. Once shocked cells are detected, the associated heating rate is estimated based on the entropy increase, providing a lower-limit measure of the energy dissipated by weak shocks into the thermal component of the ICM.

The pressure jump across a shock can be expressed as

$$\frac{\Delta P}{P} \equiv \frac{P_2 - P_1}{P_1} = \frac{2\gamma}{\gamma+1}\, y, \qquad \text{(S4)}$$

where $\gamma = 5/3$ is the adiabatic index, and P_1 and P_2 are the pre-shock and post-shock pressures, respectively. The dimensionless parameter $y = M^2 - 1$ is related to the shock Mach number M, given by

$$M = \sqrt{\frac{\rho_1 v_s^2}{\gamma P_1}}, \qquad \text{(S5)}$$

with ρ_1 the pre-shock density and v_s the shock velocity. According to the classical Rankine-Hugoniot (RH) relations, the corresponding density jump across the shock is

$$\delta_\rho \equiv \frac{\rho_2}{\rho_1} = \frac{(\gamma+1)(y+1)}{2+(\gamma-1)(y+1)}. \qquad \text{(S6)}$$

In the weak shock limit, the entropy jump across a shock can be approximated as

$$\mathrm{d}s \approx \frac{2\gamma k_B}{3(\gamma+1)^2 \mu m_H}\, y^3, \qquad \text{(S7)}$$

where μ is the mean molecular weight, m_H is the mass of a hydrogen atom, and k_B is the Boltzmann constant.

In practice, we compute the pressure and density differences between consecutive snapshots separated by $\Delta t = 1$ Myr at the same spatial grid cells. Cells are flagged as shock-heated if they simultaneously satisfy the following conditions: (i). $0.2 < \Delta P/P < 40$, which corresponds to weak shocks in the Mach number range $1.08 < M < 5.7$; (ii). $ds > 0$, ensuring the presence of local heating associated with an entropy increase; (iii). $\rho_2/\rho_1 \geq \delta_\rho$, requiring the density jump to be consistent with the RH relations, thus confirming that the flagged cells trace genuine shocked regions. For the cells flagged as shock-heated, the shock heating rate density is estimated as

$$\dot{e}_{\rm shock} = \frac{\rho_1 T_1\, ds}{\Delta t}. \qquad \text{(S8)}$$

The total shock heating rate is then obtained by summing over the shocked volume:

$$\dot{E}_{\rm shock} = \dot{e}_{\rm shock} V, \tag{S9}$$

with V denoting the corresponding cell's volume.

Different from (*18*), our shock heating estimator adopts a higher time resolution of 1 Myr, which enhances our ability to capture the contribution of strong shocks. Moreover, in condition (i) we additionally choose 40 as the upper limit for the pressure jump to ensure the validity of the weak-shock approximation used in the entropy jump equation, and to exclude the contribution of strong shocks ($\Delta P/P \gg \frac{\gamma+1}{\gamma-1}$). In condition (ii), we further impose a more stringent density jump constraint to identify shocked regions, which ensures that our estimate does not overestimate the level of shock dissipation. This procedure provides an approximate yet practical measure of the energy dissipation associated with weak shocks driven by AGN jets and winds in the ICM.

<u>KH instability driven by the jet-wind shear in the JetWind model produces the strongest turbulence among the three models</u>

The shear among jet, wind, and ICM results in Kelvin-Helmholtz (KH) instability, which can potentially grow and cause turbulence. In the following, we compare the strength of turbulence produced in the three models.

Figure S1 shows the radial velocity (v_r) profiles as a function of polar angle (θ) time-averaged over the hot mode phase for the three models. In each plot, different lines represent the radial velocity v_r at various radii ranging from 1 kpc to 30 kpc. The `JetWind` model exhibits the largest velocity gradients, indicating persistent strong shear flows throughout the entire angular domain. In contrast, the `WindOnly` simulation displays more moderate shear profiles and the `JetOnly` simulation demonstrates even flatter velocity profiles, suggesting that isolated jet feedback is considerably less effective in generating the shear flows necessary for turbulence development. For the `JetOnly` case, the cold wind launched prior to the transition to the hot accretion mode produces strong outward radial velocities in the equatorial region. As a result, a significant outflow radial velocity persists even after time-averaging over the hot-mode phase.

Based on these velocity distributions, we can estimate the growth rate of the KH instability using the relation $\gamma \sim \chi^{1/2} k v_{\rm shear}$, where χ denotes the density contrast between adjacent shear layers, k denotes the perturbation wavenumber, and $v_{\rm shear}$ corresponds to the velocity differential across the shear interface. While the density contrast χ across the angular direction is comparable among three models, the shear velocity $v_{\rm shear}$ differs substantially. In `JetWind`, $v_{\rm shear} \sim v_r$, substantially exceeding that of the `JetOnly` simulation where $v_{\rm shear} \ll v_r$. This difference results in different timescale relationships: $t_{\rm KH} \sim t_{\rm dynamic}$ in `JetWind` versus $t_{\rm KH} \gg t_{\rm dynamic}$ in `JetOnly`, where $t_{\rm dynamic} \sim r/v_r$ represents the characteristic timescale for the propagation of the outflow. Consequently, the shear flows in the `JetWind` model generate much stronger turbulence, whose dissipation leads to more efficient heating.

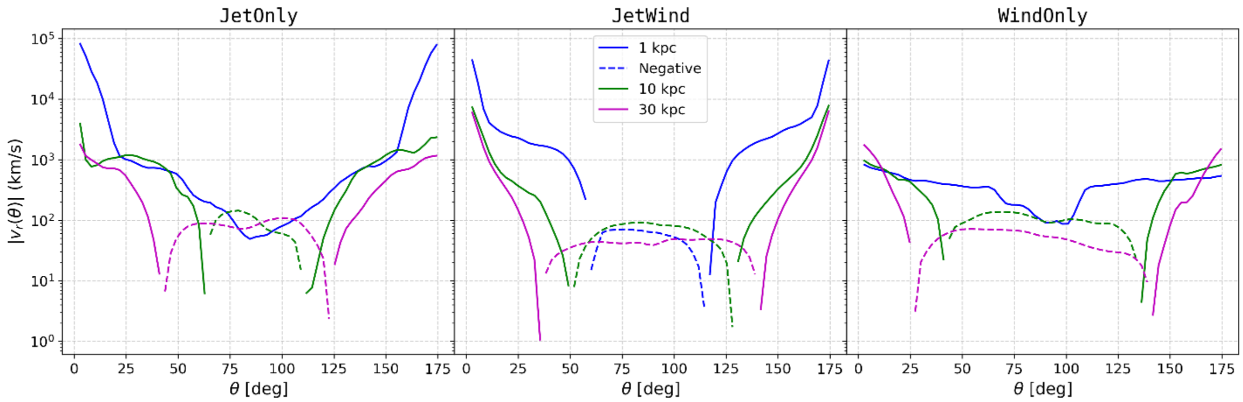

Fig. S1.

Radial velocity v_r as a function of polar angle θ, time-averaged over the hot mode phase for the three models. Different colors denote velocity profiles at various radii. The `JetWind` model exhibits the strongest velocity shear, which in turn drives the most vigorous KH instabilities and turbulence.

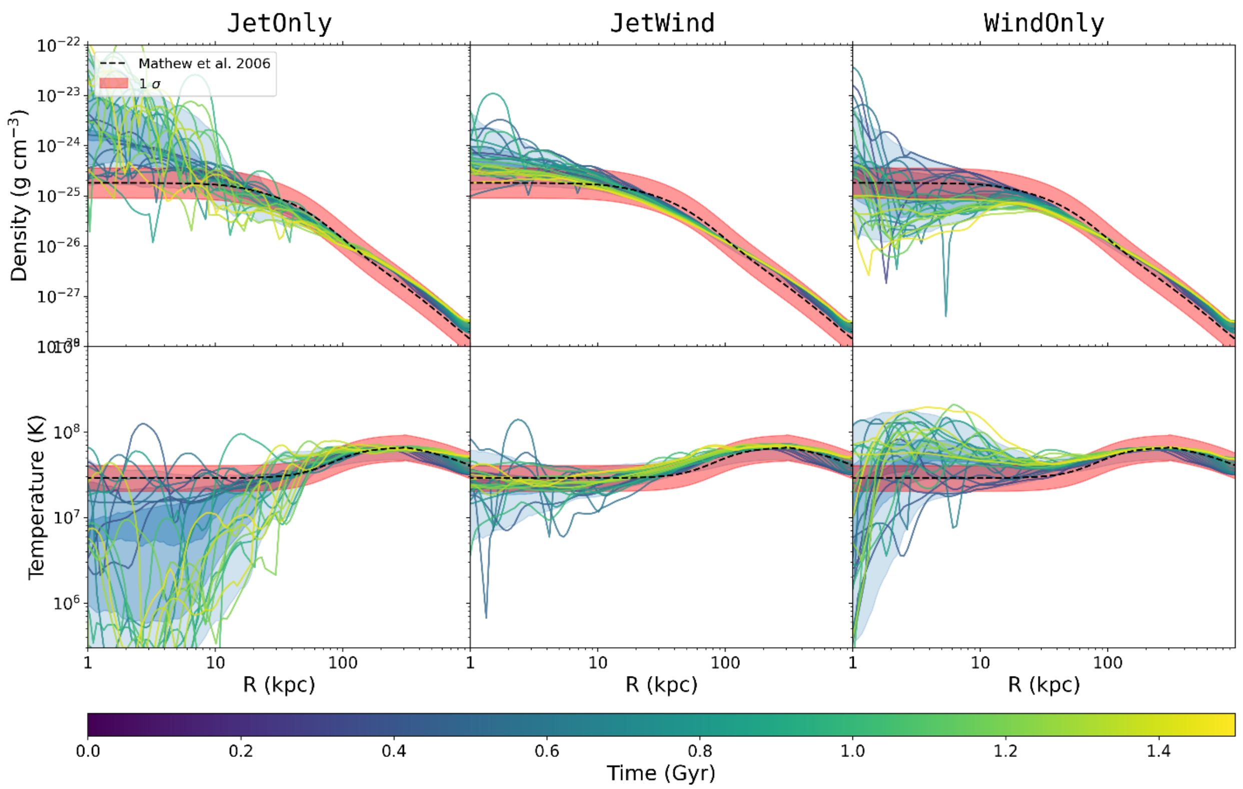

Fig. S2.

Radial profiles of the intracluster medium. Density (top) and temperature (bottom) profiles are shown for the three models, color-coded by time. Shaded areas mark the 10th–90th percentiles. The black dashed line and red shaded region shows Perseus data (*41,78*). Again, only the `JetWind` model's density and temperature profile primarily meets the observations.
